# Altered cardiorespiratory response to exercise in overweight and obese women with polycystic ovary syndrome

**DOI:** 10.14814/phy2.12719

**Published:** 2016-02-16

**Authors:** Antti‐Pekka E. Rissanen, Tiina Koskela‐Koivisto, Harriet Hägglund, Anne S. Koponen, Jyrki M. Aho, Maritta Pöyhönen‐Alho, Aila Tiitinen, Heikki O. Tikkanen, Juha E. Peltonen

**Affiliations:** ^1^ Department of Sports and Exercise MedicineClinicum, University of HelsinkiHelsinkiFinland; ^2^ Department of Obstetrics and GynecologyHelsinki University Hospital and Helsinki UniversityHelsinkiFinland; ^3^ Clinic for Sports and Exercise MedicineFoundation for Sports and Exercise MedicineHelsinkiFinland; ^4^ Institute of BiomedicineSchool of MedicineUniversity of Eastern FinlandKuopioFinland

**Keywords:** Arteriovenous oxygen difference, cardiac output, impedance cardiography, peak oxygen uptake

## Abstract

In polycystic ovary syndrome (PCOS), cardiovascular risk is increased. Peak O_2_ uptake ($\dot{V}O_{2\mathrm{peak}}$) predicts the cardiovascular risk. We were the first to examine the contribution of systemic O_2_ delivery and arteriovenous O_2_ difference to $\dot{V}O_{2\mathrm{peak}}$ in overweight and obese women with PCOS. Fifteen overweight or obese PCOS women and 15 age‐, anthropometry‐, and physical activity‐matched control women performed a maximal incremental cycling exercise test. Alveolar gas exchange (volume turbine and mass spectrometry), arterial O_2_ saturation (pulse oximetry), and cardiac output (CO) (impedance cardiography) were monitored. Hb concentration was determined. Arterial O_2_ content and arteriovenous O_2_ difference (C(a‐v)O_2_) (Fick equation) were calculated. Insulin resistance was evaluated by homeostasis model assessment (HOMA‐IR). PCOS women had lower $\dot{V}O_{2\mathrm{peak}}$ than controls (40 ± 6 vs. 46 ± 5 mL/min/kg fat‐free mass [FFM], *P *=* *0.011). Arterial O_2_ content was similarly maintained in the groups throughout the exercise test (*P *>* *0.05). Linear regression analysis revealed a pronounced response of CO to increasing $\dot{V}O_{2}$ in PCOS women during the exercise test: A ∆CO/∆$\dot{V}O_{2}$ slope was steeper in PCOS women than in controls (*β *= 5.84 vs. *β *= 5.21, *P *=* *0.004). Eventually, the groups attained similar peak CO and peak CO scaled to FFM (*P *>* *0.05). Instead, C(a‐v)O_2_ at peak exercise was lower in PCOS women than in controls (13.2 ± 1.6 vs. 14.8 ± 2.4 mL O_2_/100 mL blood, *P *=* *0.044). HOMA‐IR was similar in the groups (*P *>* *0.05). The altered cardiorespiratory responses to exercise in overweight and obese PCOS women indicate that PCOS per se is associated with alterations in peripheral adjustments to exercise rather than with limitations of systemic O_2_ delivery.

## Introduction

Polycystic ovary syndrome (PCOS) is a complex endocrinopathy characterized by chronic oligo‐anovulation, polycystic ovaries, and hyperandrogenism (Goodarzi et al. 2011). PCOS affects from 6% to 20% of women worldwide, depending on the population studied and diagnostic criteria applied (Azziz et al. 2004; Broekmans et al. 2006; Boyle et al. 2012). Hence, it is the most common endocrinopathy in reproductive‐aged women. Insulin resistance (IR) and concomitant compensatory hyperinsulinemia drive the phenotypic features of PCOS and thus are essential characteristics of the syndrome (Goodarzi et al. 2011). PCOS involves both intrinsic syndrome‐specific IR and extrinsic body mass index (BMI)‐related IR in overweight and obese women (Stepto et al. 2013). Overweight (25 kg/m^2^ ≤ BMI < 30 kg/m^2^) and obesity (BMI ≥ 30 kg/m^2^) affect over 50% of women with PCOS and exacerbate both reproductive abnormalities and metabolic dysfunction (Azziz et al. 2009).

Metabolic dysfunction in women with PCOS leads to exaggerated risk for diabetes and cardiovascular diseases with aging (The Amsterdam ESHRE/ASRM‐Sponsored 3rd PCOS Consensus Workshop Group, 2012). Peak O_2_ uptake ($\dot{V}O_{2\mathrm{peak}}$) reflects exercise capacity, which is an independent predictor of cardiovascular and all‐cause mortality in women (Mora et al. 2003). Overweight and obese women with PCOS have reduced $\dot{V}O_{2\mathrm{peak}}$ compared to age‐ and BMI‐matched healthy women (Orio et al. 2006). However, if exhibiting a similar IR profile, overweight and obese women with PCOS have been reported to have similar $\dot{V}O_{2\mathrm{peak}}$ to that of age‐ and weight‐matched healthy women (Thomson et al. 2009). Thus, the magnitude of IR has been suggested to be the leading pathophysiological feature affecting $\dot{V}O_{2\mathrm{peak}}$ in overweight and obese women with PCOS (Orio et al. 2006; Thomson et al. 2009).

$\dot{V}O_{2}$ is a product of cardiac output (CO) and systemic arteriovenous O_2_ difference (C(a‐v)O_2_). In an integrated manner, $\dot{V}O_{2}$ consists of the O_2_ transport system delivering O_2_ from the air to the muscle mitochondria and the metabolic system utilizing the delivered O_2_ to generate energy. Hence, $\dot{V}O_{2\mathrm{peak}}$ may be affected by limitations of alveolar gas exchange, Hb concentration, cardiac function, muscle blood flow, muscle O_2_ extraction, and muscle O_2_ utilization (Wagner 2000). However, little is known about mechanisms primarily determining $\dot{V}O_{2\mathrm{peak}}$ in PCOS. Both diastolic (Yarali et al. 2001; Orio et al. 2004; Kosmala et al. 2008) and systolic (Kosmala et al. 2008) dysfunction, associated with IR (Yarali et al. 2001; Orio et al. 2004; Kosmala et al. 2008), as well as normal cardiac function (Tekin et al. 2009; Rees et al. 2014) have been reported in women with PCOS at rest in case–control echocardiographic studies. Thus, cardiac dysfunction and concomitantly impaired exercise CO are potential contributors to reduced $\dot{V}O_{2\mathrm{peak}}$ in PCOS. Peripheral O_2_ delivery and utilization may also be affected in PCOS. Women with PCOS exhibit endothelial dysfunction manifested as impaired nitric oxide (NO)‐mediated vasodilation (Sprung et al. 2013a,b), which can result in impaired muscle blood flow during exercise (Maxwell et al. 1998). For the present, an association between endothelial dysfunction and IR in PCOS is unresolved (Sprung et al. 2013a). Defects in skeletal muscle insulin signaling pathways (Dunaif et al. 2001; Corbould et al. 2005) and expression of genes involved in mitochondrial oxidative metabolism (Skov et al. 2007) have also been observed in women with PCOS and pronounced IR, potentially reducing responsiveness to glucose and O_2_ utilization within skeletal muscles. However, intact primary function of skeletal muscle mitochondria has also been reported in PCOS (Eriksen et al. 2011; Rabøl et al. 2011).

Characterizing and understanding cardiorespiratory and vascular responses to exercise in women with PCOS is important as lifestyle management, including regular exercise and diet, is the first‐line strategy in pursuing health outcomes (e.g., improved ovulation and menstrual cycle regulation, reduced weight and IR) in PCOS (Harrison et al. 2011). Nevertheless, the responses of cardiac function and C(a‐v)O_2_ to exercise in women with PCOS have not yet been studied. In this study, we hypothesized that overweight and obese PCOS women would have lower $\dot{V}O_{2\mathrm{peak}}$ than control women matched for age, anthropometry, and leisure‐time physical activity (LTPA). The purpose of the study was to examine systemic O_2_ delivery (particularly cardiac function) and C(a‐v)O_2_ during incremental exercise and their contributions to the hypothetically reduced $\dot{V}O_{2\mathrm{peak}}$ of overweight and obese PCOS women.

## Materials and Methods

### Subjects

Thirty female volunteers participated in this study. Fifteen subjects were overweight or obese PCOS women, while 15 subjects were overweight or obese control women. The groups were matched for age, anthropometry, and self‐reported LTPA. All subjects were Finns and thus had a North European background. PCOS women achieved the European Society for Human Reproduction and Embryology/American Society for Reproductive Medicine criteria for PCOS diagnosis (i.e., Rotterdam criteria) (Rotterdam ESHRE/ASRM‐Sponsored PCOS Consensus Workshop Group, 2004): All showed oligo‐ or anovulation, all showed polycystic ovaries on transvaginal ultrasound, and four had clinical (hirsutism) and/or biochemical (serum testosterone > 2.0 nmol/L) hyperandrogenism. PCOS women were recruited from the patient population of the Department of Obstetrics and Gynecology, Helsinki University Hospital, Helsinki, Finland. Controls were regularly menstruating (i.e., a menstrual period occurring every 23–32 days [Cole et al. 2009]), showed no clinical evidence of hyperandrogenism, and were free of medication. Controls were mainly recruited from the employees and the students of University of Helsinki, Helsinki, Finland. The exclusion criteria of the study were an age of <18 or >40 years, BMI of <25 or >40 kg/m^2^, pregnancy, androgen‐secreting tumors, congenital adrenal hyperplasia, Cushing's syndrome, anemia, diabetes, hypertension, antiandrogen medication, *β*‐blocker medication, medication influencing glucose homeostasis, use of oral contraceptives, substance abuse, physical disability, smoking, and other cardiovascular, endocrine, musculoskeletal, neurological, or respiratory diseases that could have affected any outcome of interest.

### Ethical approval

Every subject gave written informed consent to participate in this study, which conformed to the Declaration of Helsinki and was approved by the Ethics Committee of Hospital District of Helsinki and Uusimaa, Helsinki, Finland.

### Study protocol

The subjects visited the laboratory twice. Both visits were preceded by abstinence from alcohol for at least 24 h and physical exercise for at least 12 h. At the first visit, the subjects reported to the laboratory 2–3 h after a meal. The first visit consisted of pre‐exercise measurements and a cardiorespiratory exercise test. The subjects completed a preliminary questionnaire concerning their personal health and medical history. LTPA was obtained from a single question: “If you think about your past 3 months and physical activity sessions lasting more than 20 min in all settings (e.g., commutation, walking a dog, recreation, sport), how many times a week and how long at a time have you engaged in physical activity?” The question meets the general recommendations (i.e., frequency, duration, all settings) for enquiring LTPA (van Poppel et al. 2010). The subjects’ height and waist and hip circumferences were measured, and body composition was determined by the bioimpedance method (InBody 720, Biospace Co., Ltd., Seoul, South Korea). Capillary blood was drawn from a fingertip to analyze Hb concentration by a blood gas analyzer (ABL725, Radiometer, Copenhagen, Denmark). Pre‐exercise measurements at rest also included a 12‐lead ECG, blood pressure, and flow‐volume spirometry (Medikro Spiro 2000, Medikro Oy, Kuopio, Finland). In addition, a physician examined the subjects to ensure their suitability for the cardiorespiratory exercise test, which was also performed at the first visit (see below).

The second visit followed overnight fasting. Fasting venous blood was drawn for measurements of lipid profile (i.e., HDL, LDL, and total cholesterol and triglycerides), plasma glucose, and serum insulin. Fasting plasma glucose and fasting serum insulin were determined by the hexokinase method and the immunochemiluminometric assay, respectively. Homeostasis model assessment of IR (HOMA‐IR) was calculated by the following formula (Matthews et al. 1985): HOMA‐IR = [fasting plasma glucose (mmol/L)] × [fasting serum insulin (*μ*U/mL)]/22.5.

Serum testosterone and sex hormone‐binding globulin (SHBG) concentrations of PCOS women were not determined separately for this study but as part of the patients’ clinical care by the mass spectrometry assay and the immunochemiluminometric assay, respectively. Free androgen index was calculated as follows: Free androgen index = [serum testosterone (nmol/L)]/[serum SHBG (nmol/L)] × 100.

### Cardiorespiratory exercise test

The cardiorespiratory exercise test was performed on a mechanically braked cycle ergometer (Monark Ergomedic 839E, Monark Exercise AB, Vansbro, Sweden). The test began with 5‐min seated rest while the subjects sat relaxed on the ergometer, followed by 5‐min baseline unloaded cycling. The step incremental protocol (30 W/3 min) was then initiated with a work rate of 30 W, and the subjects continued exercising until volitional fatigue. The subjects were verbally encouraged to maximal exertion.

Breath‐by‐breath ventilation was measured by a low‐resistance turbine (Triple V, Jaeger Mijnhardt, Bunnik, The Netherlands) to determine inspiratory and expiratory volumes and flow during the exercise test. Prior to each test, the turbine was calibrated using a syringe of 3.00 L volume (Hans Rudolph, Inc., Kansas City, MO). Inspired and expired gases were sampled continuously at mouth and analyzed for concentrations of O_2_, CO_2_, N_2_, and Ar by mass spectrometry (AMIS 2000, Innovision A/S, Odense, Denmark) after calibration with precision‐analyzed gas mixtures. Breath‐by‐breath respiratory data were collected as raw data. The raw data were transferred to a computer, which determined gas delays for each breath to align concentrations with volume data and to build a profile of each breath. Breath‐by‐breath alveolar gas exchange was then calculated with the AMIS algorithms, and the data were interpolated to obtain second‐by‐second values. Respiratory exchange ratio (RER) was calculated as a quotient of CO_2_ output and pulmonary O_2_ uptake ($\dot{V}O_{2}$) (Edvardsen et al. 2014). Rating of perceived exertion (RPE) was obtained using the Borg scale (6–20) at the end of each work rate.

Fingertip pulse oximetry (Nonin 9600, Nonin Medical, Inc., Plymouth, MA) was used to monitor arterial O_2_ saturation (SpO_2_). Arterial O_2_ content (CaO_2_) was calculated as the product of SpO_2_, Hb concentration, and the physiological O_2_ binding coefficient of Hb (1.34 mL/g): CaO_2_ = SpO_2_ × Hb concentration × 1.34.

Heart rate (HR) and the electrical activity of the heart were continuously monitored by ECG (PowerLab, ADInstruments, Oxford, United Kingdom). Cardiac function was continuously evaluated by an impedance cardiograph device (PhysioFlow, Manatec Biomedical, Paris, France). This method has been described in detail elsewhere (Charloux et al. 2000); briefly, the method uses changes in transthoracic impedance during cardiac ejection to calculate stroke volume (SV), which is multiplied by HR to provide an estimate of CO. The method has been found valid and reliable at rest and during low‐to‐maximal intensity exercise in overweight and obese individuals (Charloux et al. 2000; Richard et al. 2001); when CO has been measured by the PhysioFlow and the direct Fick method during maximal incremental exercise, a correlation coefficient of *r *=* *0.94 between the two methods has been observed (Richard et al. 2001). Systolic and diastolic blood pressures were measured automatically (Tango+, SunTech Medical, Morrisville, NC) from the brachial artery at seated rest and at the end of each work rate.

Mean arterial pressure (MAP) was calculated by the standard equation: MAP = (systolic blood pressure + 2 ×  diastolic blood pressure)/3. Systemic vascular resistance (SVR) was calculated according to Darcy's law: SVR = (MAP − central venous pressure)/CO, where central venous pressure was assumed to be 0 mmHg (Mortensen et al. 2005). Cardiac power output (CPO) at peak exercise was calculated from the values of CO and MAP at peak exercise (Cooke et al. 1998): CPO = CO ×MAP × *K*, where *K* is a conversion factor (2.22 × 10^−3^) into watts. Systemic C(a‐v)O_2_ was derived using the Fick equation: C(a‐v)O_2_ = $\dot{V}O_{2}$/CO.

### Statistical analysis

Data are expressed as mean ± standard deviation (SD). The mean values of the last 30 sec at seated rest, during unloaded cycling, at each work rate, and at peak exercise were chosen for further analyses. Peak O_2_ uptake ($\dot{V}O_{2\mathrm{peak}}$) was determined as the highest value of a 60‐sec moving averaging interval.

The Shapiro–Wilk test was used to check for normality and data were log transformed when appropriate. A one‐way ANOVA was used to compare baseline characteristics, cardiovascular function at seated rest, metabolic profile, cardiovascular risk factors, and CPO between PCOS women and controls. A two‐way repeated measures ANOVA was used to evaluate whether there were differences in responses to the cardiorespiratory exercise test between PCOS women and controls: Group × Exercise interactions were evaluated, while Group (i.e., PCOS women vs. controls) was a between‐subjects factor, and Exercise (i.e., unloaded cycling [0W], work rates accomplished by each subject [30 W, 60 W, 90 W, 120 W], peak exercise) was a within‐subject factor. In a separate analysis, we used relative intensities (i.e., % of $\dot{V}O_{2\mathrm{peak}}$ during unloaded cycling [0 W], 25% of $\dot{V}O_{2\mathrm{peak}}$, 50% of $\dot{V}O_{2\mathrm{peak}}$, 75% of $\dot{V}O_{2\mathrm{peak}}$, 100% of $\dot{V}O_{2\mathrm{peak}}$) as a within‐subject factor. Multivariate ANOVA with Bonferroni post hoc analysis was performed to identify the differences in the exercise responses between PCOS women and controls. Pearson's correlation coefficient was used for correlation analyses.

The response of CO to incremental exercise was determined as follows: The means of CO and $\dot{V}O_{2}$ at six data points (i.e., unloaded cycling [0 W], 30 W, 60 W, 90 W, 120 W, peak exercise) were separately calculated for PCOS women and controls, after which the ∆CO/∆$\dot{V}O_{2}$ slope for both subject groups was determined by performing a linear regression of CO over $\dot{V}O_{2}$ at the six data points. Further comparison of the ∆CO/∆$\dot{V}O_{2}$ slopes (i.e., the *β* coefficients) between PCOS women and controls was made by using the method of dummy variables and interaction terms (Lunt 2015): A dummy variable called Syndrome was first created so that it took the value 0 for controls and 1 for PCOS women. To test whether PCOS affected the linear regression of CO over $\dot{V}O_{2}$, an interaction term Syndrome × $\dot{V}O_{2}$ was created and then included in an additional multiple linear regression model, where CO was the outcome, and Syndrome, $\dot{V}O_{2}$, and Syndrome × $\dot{V}O_{2}$ were the predictors. Eventually, the contribution of Syndrome × $\dot{V}O_{2}$ to the model indicated whether the ∆CO/∆$\dot{V}O_{2}$ slopes differed between PCOS women and controls.

To avoid ignoring any differences in body size and composition between PCOS women and controls, we scaled $\dot{V}O_{2}$ (Batterham et al. 1999; Krachler et al. 2015) as well as SV, CO, and CPO (Whalley et al. 2004) relative to fat‐free mass (FFM), whereas SVR was multiplied by FFM. The scaled cardiovascular variables were referred as indices: SV_i_, CO_i_, CPO_i_, SVR_i_, respectively.

Statistical significance was set at a *P* value of <0.05. The results were computed with PASW Statistics 18.0 (IBM Corporation, Somers, NY).

## Results

### Descriptive characteristics of the subjects

Baseline characteristics, cardiovascular function at seated rest, metabolic profile, and cardiovascular risk factors of the subjects are summarized in Tables 1 and 2. PCOS women and controls had similar Hb concentrations and flow‐volume spirometry results with no defects. No differences between the groups were observed for systolic and diastolic blood pressures, HR, SV, and SV_i_ at rest. At rest, PCOS women had higher CO, but similar CO_i_ in comparison with controls. Fasting glucose, fasting insulin, HOMA‐IR, LDL cholesterol, total cholesterol, and triglycerides were similar between the groups. PCOS women had lower HDL cholesterol than controls. In PCOS women, serum testosterone was 1.4 ± 0.7 nmol/L, serum SHBG was 40 ± 17 nmol/L, and free androgen index was 4.3 ± 4.0.

**Table 1:** Baseline characteristics of the subjects and cardiovascular function at seated rest

	PCOS (*n *=* *15)	Controls (*n *=* *15)	*P*
Baseline characteristics			
Age (years)	29.3 ± 4.0	31.1 ± 5.5	0.326
Height (cm)	171 ± 6	167 ± 10	0.166
Weight (kg)	94 ± 8	86 ± 17	0.121
Body mass index (kg/m^2^)	32.0 ± 2.0	30.6 ± 3.9	0.208
Body fat (%)	41 ± 4	39 ± 6	0.386
Fat‐free mass (kg)	55 ± 5	52 ± 8	0.106
Waist‐to‐hip ratio	0.86 ± 0.07	0.83 ± 0.08	0.279
Leisure‐time physical activity (h:min/wk)	2:22 ± 1:02	2:33 ± 1:19	0.691
Hb concentration (g/L)	132 ± 9	134 ± 7	0.685
FVC (L)	4.2 ± 0.4	4.3 ± 0.7	0.743
FVC (% of reference value)	96 ± 8	103 ± 14	0.105
FEV_1_ (L)	3.5 ± 0.4	3.4 ± 0.5	0.623
FEV_1_ (% of reference value)	92 ± 8	95 ± 11	0.352
Cardiovascular function at seated rest			
Systolic blood pressure (mmHg)	116 ± 8	116 ± 17	0.979
Diastolic blood pressure (mmHg)	82 ± 8	76 ± 10	0.078
HR (bpm)	86 ± 12	79 ± 12	0.120
SV (mL)	73 ± 11	66 ± 12	0.073
SV_i_ (mL/kg FFM)	1.33 ± 0.22	1.29 ± 0.25	0.655
CO (L/min)	6.2 ± 0.8	5.2 ± 1.1	0.006
CO_i_ (mL/min/kg FFM)	114 ± 20	101 ± 18	0.071

Data are means ± SD.

FVC, forced vital capacity; FEV_1_, forced expiratory volume in 1 sec; HR, heart rate; SV, stroke volume; SV_i_, stroke volume index; FFM, fat‐free mass; CO, cardiac output; CO_i_, cardiac output index.

**Table 2:** Metabolic profile and cardiovascular risk factors of the subjects

	PCOS (*n *=* *15)	Controls (*n *=* *15)	*P*
Fasting glucose (mmol/L)	5.4 ± 0.4^a^	5.5 ± 0.6	0.814
Fasting insulin (*μ*U/mL)	12.1 ± 6.9^a^	10.0 ± 4.4	0.356
HOMA‐IR	3.0 ± 1.8^a^	2.5 ± 1.3	0.442
HDL cholesterol (mmol/L)	1.3 ± 0.3	1.6 ± 0.4	0.043
LDL cholesterol (mmol/L)^b^	3.0 ± 0.8	3.0 ± 0.9	0.764
Total cholesterol (mmol/L)	4.6 ± 0.7	4.7 ± 0.9	0.604
Triglycerides (mmol/L)	1.1 ± 0.5	1.0 ± 0.4	0.496

Data are means ± SD.

a *n *=* *14.

b Log transformed for statistical analysis due to non‐normally distributed data.

HOMA‐IR, homeostasis model assessment index of insulin resistance.

### Responses to cardiorespiratory exercise test

**Figure 1:**
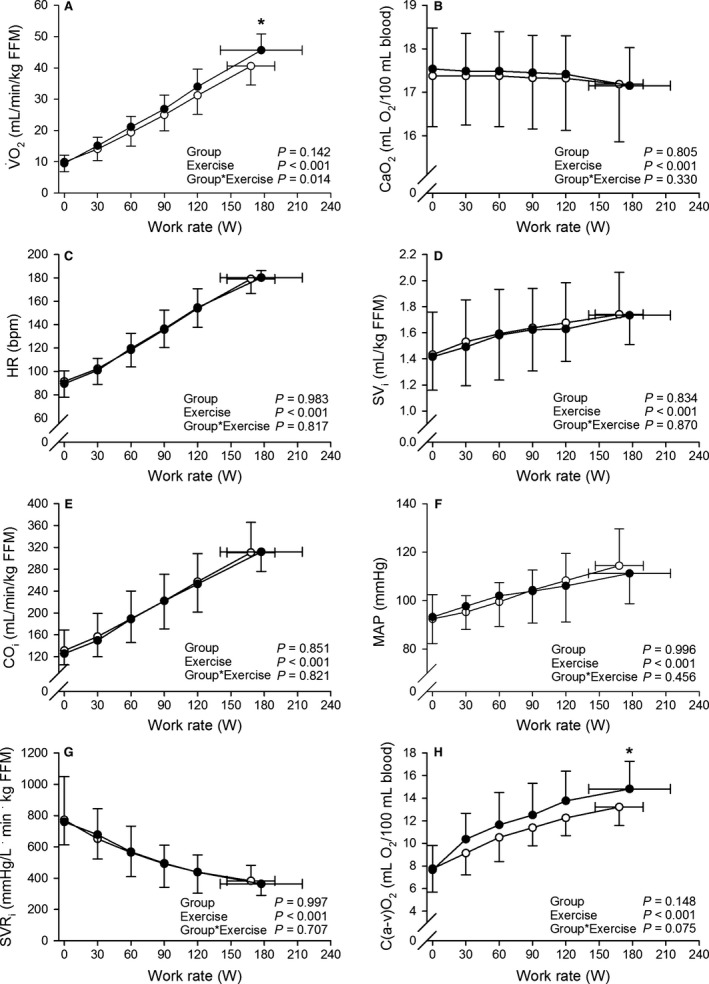
Pulmonary O_2_ uptake scaled relative to fat‐free mass ($\dot{V}O_{2}$) (A), arterial O_2_ content (CaO_2_) (B), heart rate (HR) (C), stroke volume index (SV_i_) (D), cardiac output index (CO_i_) (E), mean arterial pressure (MAP) (F), systemic vascular resistance index (SVR_i_) (G), and arteriovenous O_2_ difference (C(a‐v)O_2_) (H), as a function of work rate. White circles (○) = PCOS women (*n *=* *15), black circles (●) = controls (*n *=* *15). The *P* values refer to a two‐way repeated measures ANOVA: Group × Exercise interaction is evaluated, while Group (PCOS women vs. controls) is a between‐subjects factor and Exercise (unloaded cycling [0 W], work rates accomplished by each subject [30 W, 60 W, 90 W, 120 W], peak exercise) is a within‐subject factor. *Post hoc significantly different from PCOS women at *P *<* *0.05.

**Figure 2:**
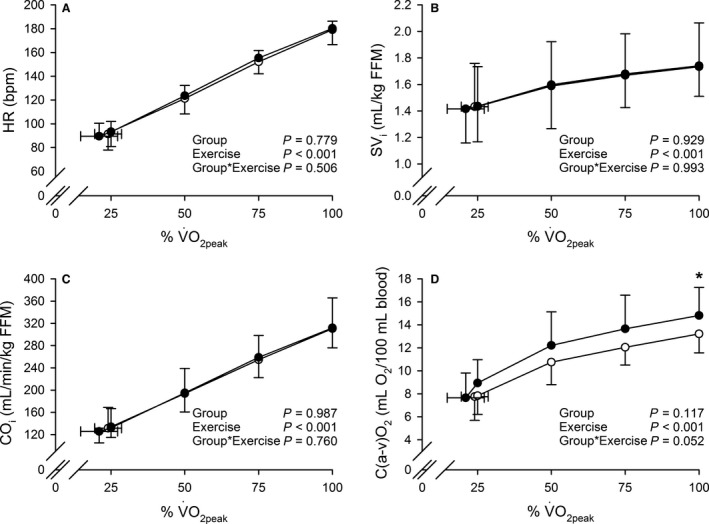
Heart rate (HR) (A), stroke volume index (SV_i_) (B), cardiac output index (CO_i_) (C), and arteriovenous O_2_ difference (C(a‐v)O_2_) (D), as a function of relative intensity (% of peak pulmonary O_2_ uptake [$\dot{V}O_{2\mathrm{peak}}$]). White circles (○) = PCOS women (*n *=* *15), black circles (●) = controls (*n *=* *15). The *P* values refer to a two‐way repeated measures ANOVA: Group × Exercise interaction is evaluated, while Group (PCOS women vs. controls) is a between‐subjects factor and Exercise (% of $\dot{V}O_{2\mathrm{peak}}$ during unloaded cycling [0 W], 25% of $\dot{V}O_{2\mathrm{peak}}$, 50% of $\dot{V}O_{2\mathrm{peak}}$, 75% of $\dot{V}O_{2\mathrm{peak}}$, 100% of $\dot{V}O_{2\mathrm{peak}}$) is a within‐subject factor. *Post hoc significantly different from PCOS women at *P *<* *0.05.

$\dot{V}O_{2}$ and cardiovascular responses to the incremental exercise test are presented in Figures 1 and 2. Table 3 details peak work rates as well as respiratory and cardiovascular responses at peak exercise. A significant Group × Exercise interaction was observed for $\dot{V}O_{2}$ (*P *=* *0.014; Fig. 1A). When scaled to FFM, peak work rate and $\dot{V}O_{2\mathrm{peak}}$ were lower in PCOS women than in controls, while the differences in absolute values were not significant. At peak exercise, RER (1.12 ± 0.05 vs. 1.12 ± 0.05, *P *=* *0.845) and RPE (20 ± 1 vs. 19 ± 1, *P *=* *0.346) were similar between PCOS women and controls, respectively, indicating that both groups similarly made their maximal effort during the exercise test.

**Table 3:** Work rates and respiratory and cardiovascular responses at peak exercise

	PCOS (*n *=* *15)	Controls (*n *=* *15)	*P*
Work rate (W)	168 ± 22	177 ± 37	0.406
Work rate (W/kg FFM)	3.0 ± 0.3	3.5 ± 0.5	0.015
$\dot{V}O_{2}$ (L/min)	2.22 ± 0.27	2.36 ± 0.39	0.264
$\dot{V}O_{2}$ (mL/min/kg)	24 ± 3	28 ± 5	0.006
$\dot{V}O_{2}$ (mL/min/kg FFM)	40 ± 6	46 ± 5	0.011
Ventilation (L/min)	89.5 ± 7.5	96.6 ± 16.3	0.136
SpO_2_ (%)	97 ± 2	96 ± 2	0.185
CaO_2_ (mL O_2_/100 mL blood)	17.2 ± 1.3	17.2 ± 0.9	0.933
HR (bpm)	179 ± 12	180 ± 6	0.768
SV (mL)	95 ± 13	89 ± 15	0.224
SV_i_ (mL/kg FFM)	1.74 ± 0.32	1.73 ± 0.22	0.945
CO (L/min)	17.0 ± 2.2	16.0 ± 2.7	0.259
CO_i_ (mL/min/kg FFM)	310 ± 55	312 ± 36	0.941
Systolic blood pressure (mmHg)	176 ± 18	167 ± 21	0.231
Diastolic blood pressure (mmHg)	83 ± 17	87 ± 13	0.943
MAP (mmHg)	114 ± 15	111 ± 12	0.537
SVR (mmHg/L∙min)	6.8 ± 1.4	7.1 ± 1.4	0.587
SVR_i_ (mmHg/L∙min∙kg FFM)	383 ± 99	363 ± 74	0.543
C(a‐v)O_2_ (mL O_2_/100 mL blood)	13.2 ± 1.6	14.8 ± 2.4	0.044
CPO (W)	4.3 ± 0.7	4.0 ± 0.8	0.213
CPO_i_ (W/FFM)	0.08 ± 0.01	0.08 ± 0.01	0.733

Data are means ± SD.

FFM, fat‐free mass; $\dot{V}O_{2}$, pulmonary O_2_ uptake; SpO_2_, arterial O_2_ saturation; CaO_2_, arterial O_2_ content; HR, heart rate; SV, stroke volume; SV_i_, stroke volume index; CO, cardiac output; CO_i_, cardiac output index; MAP, mean arterial pressure; SVR, systemic vascular resistance; SVR_i_, systemic vascular resistance index; C(a‐v)O_2_, systemic arteriovenous O_2_ difference; CPO, cardiac power output; CPO_i_, cardiac power output index.

No difference between PCOS women and controls were observed for ventilation at peak exercise. SpO_2_ and CaO_2_ were also similarly maintained in the groups throughout the exercise test.

Similar profiles of HR, SV_i_, CO_i_, MAP, and SVR_i_ as a function of work rate, and similar profiles of HR, SV_i_, and CO_i_ as a function of relative intensity (% of $\dot{V}O_{2\mathrm{peak}}$) were seen in PCOS women and controls throughout the exercise: Significant Group×Exercise interactions in these analyses were not observed. In addition, similar HR, SV, SV_i_, CO, CO_i_, systolic blood pressure, diastolic blood pressure, MAP, SVR, SVR_i_, CPO, and CPO_i_ were detected in the groups at peak exercise.

**Figure 3:**
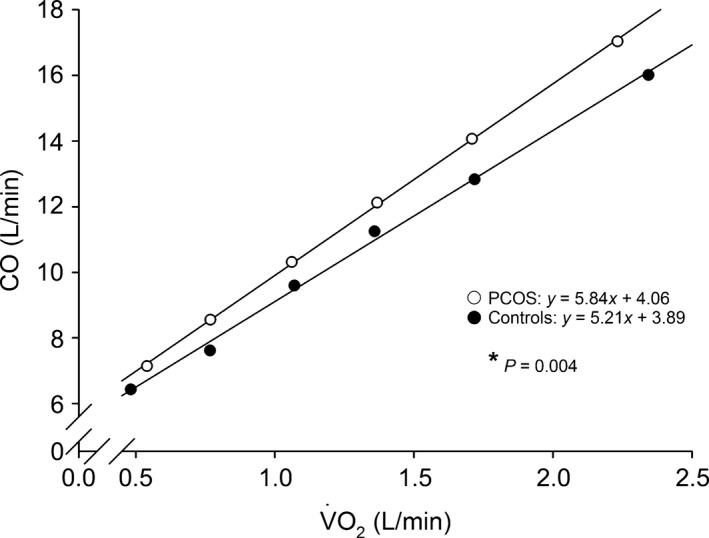
Linear regression lines presenting mean responses of cardiac output (CO) to increasing pulmonary O_2_ uptake ($\dot{V}O_{2}$) during incremental exercise. White circles (○) = PCOS women (*n *=* *15), black circles (●) = controls (*n *=* *15). The six data points for both PCOS women and controls indicate the group means of CO and $\dot{V}O_{2}$ during unloaded cycling (0 W), at work rates of 30 W, 60 W, 90 W, and 120 W, and at peak exercise. The ∆CO/∆$\dot{V}O_{2}$ slopes for both groups have been determined by performing a linear regression of CO over $\dot{V}O_{2}$ at the six data points. *The *P* value refers to the significantly different ∆CO/∆$\dot{V}O_{2}$ slopes between PCOS women (*β *= 5.84) and controls (*β *= 5.21).

Hyperbolic responses of C(a‐v)O_2_ to incremental exercise were seen in PCOS women and controls (Figs. 1H and 2D). Group × Exercise interactions for C(a‐v)O_2_ (*P *=* *0.075 when examining C(a‐v)O_2_ as a function of work rate, and *P *=* *0.052 when examining C(a‐v)O_2_ as a function of % of $\dot{V}O_{2\mathrm{peak}}$; Figs. 1H and 2D) suggested there might be true group effects at single work rates or relative intensities regarding C(a‐v)O_2_. Consequently, PCOS women had lower C(a‐v)O_2_ at peak exercise than controls (Figs. 1H and 2D, Table 3). Additionally, the ∆CO/∆$\dot{V}O_{2}$ slope was steeper in PCOS women when compared to controls (*β *= 5.84 vs. *β *= 5.21, respectively, *P *=* *0.004; Fig. 3).

In all subjects, an inverse correlation between $\dot{V}O_{2\mathrm{peak}}$ (mL/min/kg FFM) and HOMA‐IR was observed, but it did not reach statistical significance (*r *=* *−0.29, *P *=* *0.066). Instead, when $\dot{V}O_{2\mathrm{peak}}$ was scaled relative to body weight, there was a significant correlation between $\dot{V}O_{2\mathrm{peak}}$ and HOMA‐IR in all subjects (*r *=* *−0.34, *P *=* *0.037). When the correlation between $\dot{V}O_{2\mathrm{peak}}$ (mL/min/kg FFM) and HOMA‐IR was examined separately in PCOS women and controls, no significant correlation cofficients were observed: *r *=* *−0.29 (*P *=* *0.155) in PCOS women and *r *=* *−0.19 (*P *=* *0.247) in controls.

## Discussion

According to our hypothesis, reduced $\dot{V}O_{2\mathrm{peak}}$ was observed in overweight and obese women with PCOS when compared to that of age‐, anthropometry‐, and LTPA‐matched controls. To explain this, two novel findings were made. First, reduced C(a‐v)O_2_ at peak exercise and a steeper ∆CO/∆$\dot{V}O_{2}$ slope in PCOS women reflected an altered response to incremental exercise in comparison with controls. Second, PCOS women and controls attained similar cardiac function at peak exercise, while CaO_2_ was similarly maintained in the groups throughout the exercise. These findings in the relatively healthy overweight and obese women indicate that PCOS per se is associated with alterations in peripheral adjustments to exercise rather than with limitations of systemic O_2_ delivery.

### Altered cardiorespiratory response to exercise in PCOS women: reduced $\dot{V}O_{2\mathrm{peak}}$, reduced C(a‐v)O_2_ at peak exercise, steeper ∆CO/∆$\dot{V}O_{2}$ slope

We observed lower $\dot{V}O_{2\mathrm{peak}}$ in PCOS women than in controls, while there was no significant difference in HOMA‐IR between the groups. Previously, reduced $\dot{V}O_{2\mathrm{peak}}$ in overweight and obese women with PCOS has been reported when compared to age‐ and BMI‐matched healthy women (Orio et al. 2006), but if exhibiting a similar IR profile, obese women with PCOS have been found to have similar $\dot{V}O_{2\mathrm{peak}}$ to that of age‐ and weight‐matched healthy women (Thomson et al. 2009). In addition, an inverse association between $\dot{V}O_{2\mathrm{peak}}$ and HOMA‐IR has been reported in women with PCOS (Orio et al. 2006) and in a pooled population including obese women with and without PCOS (Thomson et al. 2009). Hence, it has been hypothesized that IR would be significantly linked to $\dot{V}O_{2\mathrm{peak}}$ in overweight and obese women with PCOS (Orio et al. 2006; Thomson et al. 2009). However, $\dot{V}O_{2\mathrm{peak}}$ has been scaled to body weight in the two studies (Orio et al. 2006; Thomson et al. 2009), which ignores body composition and thus may introduce a bias against overweight and obese women (Krachler et al. 2015) as discussed later. An inverse association between FFM‐adjusted $\dot{V}O_{2\mathrm{peak}}$ (mL/min/kg FFM) and HOMA‐IR did not reach statistical significance in our study. Instead, when $\dot{V}O_{2\mathrm{peak}}$ was scaled to body weight, the association between $\dot{V}O_{2\mathrm{peak}}$ and HOMA‐IR in all subjects turned out significant. Taken together, it seems that IR is likely linked to the reduced $\dot{V}O_{2\mathrm{peak}}$ in overweight and obese women with PCOS but the link becomes weaker when the confounding effect of adipose tissue is taken into account. This suggests that other pathophysiological features, not related to IR, also have a significant impact on $\dot{V}O_{2\mathrm{peak}}$ in women with PCOS.

In addition to the reduced $\dot{V}O_{2\mathrm{peak}}$, we observed both reduced C(a‐v)O_2_ at peak exercise and a steeper ∆CO/∆$\dot{V}O_{2}$ slope in PCOS women when compared to controls. C(a‐v)O_2_ also tended to be lower in PCOS women than in controls throughout the exercise until reaching the significant difference at peak exercise (Figs. 1H and 2D). These findings indicate that PCOS women exhibited a pronounced response of CO to increasing O_2_ demand from the very beginning of incremental exercise to compensate for alterations in peripheral adjustments to exercise.

We hypothesize that following alterations regarding peripheral O_2_ delivery and utilization may be associated with the reduced peak C(a‐v)O_2_ and the steeper ∆CO/∆$\dot{V}O_{2}$ slope in PCOS women. First, endothelial dysfunction, which is an intrinsic feature of PCOS (Sprung et al. 2013a), is associated with reduced exercise capacity in women (Patel et al. 2005). Endothelial dysfunction may impair the appropriate exercise‐induced hyperemia in active muscles due to reduced bioavailability of NO (Maxwell et al. 1998), and in fact, particularly NO‐mediated microvascular vasodilator dysfunction has recently been observed in PCOS (Sprung et al. 2013b). In addition, exercise training has been demonstrated to enhance NO‐mediated endothelial function with a parallel improvement in $\dot{V}O_{2\mathrm{peak}}$ in obese women with PCOS (Sprung et al. 2013b). For the present, it is unresolved whether endothelial dysfunction is associated with IR in PCOS (Sprung et al. 2013a). We did not observe differences in exercise MAP or SVR_i_ between PCOS women and controls in our current study. This suggests vascular conductance was not limited at the whole‐body level for PCOS women. However, we cannot draw conclusions concerning exercise‐induced redistribution of blood flow between active muscles and less active regions such as splanchnic circulation, because local active muscle blood flow differs considerably from systemically observed circulatory responses during incremental exercise (Murias et al. 2013). Second, in conditions in which there is a skeletal muscle oxidative defect, such as in mitochondrial myopathies, the findings of reduced $\dot{V}O_{2\mathrm{peak}}$, reduced peak C(a‐v)O_2_, and a pronounced ∆CO/∆$\dot{V}O_{2}$ slope resemble our findings in PCOS women (Taivassalo et al. 2003). Defects in skeletal muscle insulin signaling pathways (Dunaif et al. 2001; Corbould et al. 2005) and expression of genes involved in mitochondrial oxidative metabolism (Skov et al. 2007) have been observed in women with PCOS and pronounced IR, potentially reducing responsiveness to glucose and O_2_ utilization within skeletal muscles. However, primary function of skeletal muscle mitochondria has also been reported to be intact in PCOS (Eriksen et al. 2011; Rabøl et al. 2011). Hence, it is unclear whether defects in mitochondrial function could reduce O_2_ utilization and thus peak C(a‐v)O_2_ in PCOS women in this study.

### PCOS per se does not limit systemic O_2_ delivery during exercise

SpO_2_ and CaO_2_ were similarly maintained in PCOS women and controls throughout incremental exercise, suggesting that alveolar gas exchange set no limitation to systemic O_2_ delivery. In addition, after exhibiting the pronounced response of CO to increasing O_2_ demand, PCOS women attained similar CO and CO_i_ at peak exercise in comparison with controls. We also calculated CPO (and CPO_i_), which is an index of cardiac reserve and conveys the hydraulic power of the heart by relating changes in flow and afterload (Cooke et al. 1998): No differences between PCOS women and controls were observed for CPO and CPO_i_ at peak exercise. Thus, while cardiac function has its vital role in the integrated system responding for O_2_ transport (Wagner 2000), total blood flow and cardiovascular system's ability to generate an appropriate response to increasing O_2_ demand were not further diminished in PCOS women. In conclusion, PCOS per se did not contribute to limitations of systemic O_2_ delivery during exercise.

At rest before incremental exercise, higher CO but similar CO_i_ were measured in PCOS women when compared to controls. The difference in absolute CO at rest was thus partly due to slight but nonsignificant differences in anthropometry but also due to a nonsignificantly higher mean of resting HR in PCOS women. Previous studies have reported both diastolic (Yarali et al. 2001; Orio et al. 2004; Kosmala et al. 2008) and systolic (Kosmala et al. 2008) dysfunction as well as normal cardiac function (Tekin et al. 2009; Rees et al. 2014) in women with PCOS at rest in case–control echocardiographic studies. There may be an inverse association between cardiac function and the magnitude of IR in PCOS as associations between cardiac dysfunction and IR have been observed in women with PCOS (Yarali et al. 2001; Orio et al. 2004; Kosmala et al. 2008). In addition, diastolic dysfunction was recently found to be associated with IR in overweight and obese women but PCOS itself did not contribute to the dysfunction (Rees et al. 2014). Thus, our finding of similar cardiac function at peak exercise in PCOS women and controls might be due to rather similar HOMA‐IR in the groups, albeit we did not find significant associations between cardiac function and HOMA‐IR (data not shown).

### Methodological considerations, strengths, and limitations

The contribution of fat mass to O_2_ consumption is negligible and independent of $\dot{V}O_{2\mathrm{peak}}$ (Goran et al. 2000). Instead, absolute $\dot{V}O_{2\mathrm{peak}}$ (L/min) follows a linear function of FFM (Batterham et al. 1999; Krachler et al. 2015). Scaling $\dot{V}O_{2\mathrm{peak}}$ to FFM during cycling exercise was recently demonstrated to introduce a much smaller bias against obese women than quantifying $\dot{V}O_{2\mathrm{peak}}$ with more traditional methods (i.e., L/min, or mL/min/kg body weight) (Krachler et al. 2015), although cycling is a nonweight‐bearing exercise mode. Thus, in our current study involving overweight and obese women, we scaled $\dot{V}O_{2}$ to FFM to avoid ignoring any differences in body size and composition between PCOS women and controls.

The strengths of this study included matching the subject groups for age, anthropometry, and LTPA. Moreover, compared to invasive alternatives, the noninvasive methods used to monitor SpO_2_ and cardiac function were probably more feasible to the subjects of this study. This is relevant as a confidence in the ability to maintain exercise might be impaired in PCOS due to diminished mental health often related to the syndrome (Banting et al. 2014). However, PCOS women and controls similarly made their maximal effort during the exercise test according to RER and RPE data (Edvardsen et al. 2014), thus indicating no particular signs of behavior affecting their exercise performance.

One limitation of this study was the method used to assess IR; using an oral glucose tolerance test or a euglycemic–hyperinsulinemic clamp technique might have provided a more in‐depth assessment of insulin sensitivity. It is also notable that the hormonal profile of the subjects was not determined separately for this study because previous studies had not found consistent associations between hormonal profile and $\dot{V}O_{2\mathrm{peak}}$ (Orio et al. 2006; Thomson et al. 2009) or hormonal profile and cardiac function (Yarali et al. 2001; Orio et al. 2004; Rees et al. 2014) in women with PCOS. It may be suggested that hyperandrogenism did not have a major effect on the findings of this study as only 27% (4/15) of PCOS women had evidence of hyperandrogenism and androgen levels in PCOS women were relatively low. However, the impact of severe hyperandrogenism on exercise responses in women with PCOS cannot be evaluated based on this study. The amount of LTPA was determined by self‐reporting, which is notable as overweight and obese individuals may overreport the amount of their physical activity (Prince et al. 2008). However, possible overreporting hardly affected matching our subject groups for LTPA and thus the conclusions drawn. A fairly small sample size also limits this study by affecting the statistical power of the results, highlighted in correlation analyses. However, we had the statistical power of 84.5% (*P *<* *0.05) to detect the difference in our main outcome ($\dot{V}O_{2\mathrm{peak}}$) between PCOS women and controls, indicating the difference did not result from type I error.

## Conclusions

Performing maximal incremental cycling exercise revealed reduced $\dot{V}O_{2\mathrm{peak}}$, reduced C(a‐v)O_2_ at peak exercise, and a steeper ∆CO/∆$\dot{V}O_{2}$ slope in PCOS women when compared to control women matched for age, anthropometry, and physical activity. This can be regarded as an altered cardiorespiratory response to exercise in PCOS women. Eventually, PCOS women and controls attained similar cardiac function at peak exercise, while CaO_2_ was similarly maintained in the groups. In summary, these findings indicate that PCOS per se is associated with alterations in peripheral adjustments to exercise rather than with limitations of systemic O_2_ delivery in overweight and obese women. Future studies are warranted to identify detailed mechanisms of the altered peripheral adjustments indicated here.

## Conflict of Interests

None declared.
